# Resource-rich Intensive Care Units vs. Standard Intensive Care Units on Patient Mortality: A Nationwide Inpatient Database Study

**DOI:** 10.31662/jmaj.2021-0098

**Published:** 2021-09-27

**Authors:** Hiroyuki Ohbe, Yusuke Sasabuchi, Hiroki Matsui, Kiyohide Fushimi, Hideo Yasunaga

**Affiliations:** 1 Department of Clinical Epidemiology and Health Economics, School of Public Health, The University of Tokyo, Tokyo, Japan; 2 Data Science Center, Jichi Medical University, Shimotsuke, Japan; 3 Department of Health Policy and Informatics, Tokyo Medical and Dental University Graduate School of Medicine, Tokyo, Japan

**Keywords:** intensive care unit, intensivist, administrative database

## Abstract

**Introduction:**

In this present study, we aimed to assess whether care in resource-rich intensive care unit (ICU) was associated with lower ICU mortality compared with care in standard ICU.

**Methods:**

This retrospective cohort study used administrative data that are routinely collected in Japan. Using the Japanese Diagnosis Procedure Combination inpatient database, we identified patients aged >15 years who were admitted to the ICU from April 2016 to March 2019. We defined resource-rich ICUs as ICUs with two or more intensivists as full-time employees, ≥20 m^2^ per ICU bed, and a medical engineer in the hospital 24 hours per day; other ICUs were categorized as standard ICUs. The primary outcome was ICU mortality. A generalized estimating equation approach with ICUs as the clusters was used to compare ICU mortality between the two groups.

**Results:**

Of the 789,630 eligible patients from 458 ICUs, 237,138 (30%) were treated in the 111 resource-rich ICUs, whereas 552,492 (70%) were treated in the 347 standard ICUs. The crude ICU mortality rate was 3.6% (8443/237,138) among patients admitted to resource-rich ICUs, while it was 4.3% (23,490/552,492) among those admitted to standard ICUs. The results of the generalized estimating equation analysis showed that patients treated in resource-rich ICUs tended to have lower ICU mortality compared to patients treated in standard ICUs (difference, −0.4%; 95% confidence interval, −0.8%-0.0%).

**Conclusions:**

The findings of this nationwide study suggest that, compared with care in standard ICUs, care in resource-rich ICUs is associated with lower ICU mortality. This study showed the overall effect of treatment in hospitals with resource-rich ICUs including intensivist staffing and greater hospital resources. Further studies are required to assess the effects of organizational factors on mortality.

## Introduction

Outcomes for critically ill patients vary widely in different intensive care units (ICUs) ^(1), (2)^, as may be affected by organizational factors including intensivist staffing ^(3), (4), (5)^, whether the ICU is closed or open ^(6), (7)^, hospital and ICU patient volume ^(8), (9)^, and nurse staffing patterns ^(10)^.

In April 2014, Japan’s National Health Insurance System adopted a new medical fee system. Under this new system, the medical reimbursement for resource-rich ICUs (i.e., those that have two or more intensivists as full-time workers, ≥20 m^2^ per ICU bed, and a medical engineer in the hospital 24 hours per day) is 1.5 times more than that for other ICUs ^(11)^. Many Japanese hospitals have attempted to qualify as resource-rich ICUs by recruiting intensivists and improving their equipment, but only about 30% of all ICUs met these criteria in 2017, mainly because of a shortage of intensivists ^(12), (13), (14), (15), (16)^.

Although critical care resources have been shown to improve patients’ prognosis ^(3), (4), (5), (6), (7), (8), (9), (10)^, no studies have evaluated whether critically ill patients should be treated in resource-rich ICUs or standard ICUs in Japan. Therefore, this present study aimed to compare mortality between patients cared for in resource-rich ICUs vs. those cared for in standard ICUs, using a nationwide inpatient database in Japan.

## Material and Methods

This retrospective cohort study used administrative data that are routinely collected in Japan. The Institutional Review Board of the University of Tokyo approved this study (approval number 3501-3; December 25, 2017). No information allowing the identification of individual patients, hospitals, or physicians was obtained, and the requirement for informed consent was waived because of the anonymous nature of the data.

### Data source

We used the Japanese Diagnosis Procedure Combination inpatient database, which contains discharge abstracts and administrative claims data from more than 1,200 acute-care hospitals in Japan that voluntarily contribute to the database ^(17)^. This database includes the following patient-level data for all hospitalizations: age; sex; diagnoses recoded with *International Classification of Diseases, Tenth Revision* codes; daily procedures recorded using Japanese medical procedure codes; daily drug administrations; and admission and discharge status. A previous validation study of this database has showed high specificity and moderate sensitivity of the diagnoses, as well as high specificity and high sensitivity of the procedures ^(18)^.

We also used facility information and statistics presented in the Survey of Medical Institutions 2017 ^(12)^. In October 2017, there were 629 ICU-equipped hospitals with 7,109 ICU beds in Japan, corresponding to an estimated 5.6 ICU beds per 100,000 persons in the country ^(12)^. The Survey of Medical Institutions includes hospital zip code, types of wards (either general or ICU), whether the institution is a teaching hospital, whether the institution is an academic hospital, number of hospital beds in each ward, and number of dedicated nurses, pharmacists, and physical therapists working as full-time employees in each ward. We then combined this information with the data in the Japanese Diagnosis Procedure Combination inpatient database using a specific hospital identifier.

### Study population

We identified all patients aged >15 years in the Japanese Diagnosis Procedure Combination inpatient database who were admitted to the ICU from April 2016 to March 2019. Although data since April 2014 were made available, we opted to not use the data from the first 2 years because this period was before the establishment of the new medical reimbursement system for ICUs. We excluded patients aged ≤15 years because these patients are admitted to pediatric ICUs in Japan, and there is no distinction between standard and resource-rich pediatric ICUs. We excluded patients from hospitals that could not be combined with the data from the Survey of Medical Institutions 2017. When patients entered the ICU more than once during a single admission, we only included the first ICU admission.

As per the Japanese National Health Insurance System, an ICU is defined as a separate unit providing critical care services, with at least one physician on site 24 hours per day, around-the-clock nursing, the equipment necessary to care for critically ill patients, and a nurse-to-patient ratio of 1:2. In this present study, ICUs that had two or more intensivists working as full-time employees, ≥20 m^2^ per ICU bed, and a medical engineer in the hospital 24 hours per day were defined as resource-rich ICUs ^(11)^, and the remaining ICUs were defined as standard ICUs. The definition of an intensivist in the Japanese National Health Insurance System is a physician who (i) has at least 5 years of experience in critical care, (ii) has attended at least 30 hours of coursework related to critical care, and (iii) is qualified to apply for the position of certified intensivist through the Japanese Society of Intensive Care Medicine. The Japanese procedure codes used to define resource-rich ICUs and standard ICUs are presented in Supplementary Table 1.

### Outcome and covariates

The primary outcome was ICU mortality. Covariates were age, sex, body mass index at hospital admission, Japan Coma Scale at hospital admission ^(19)^, Charlson Comorbidity Index score ^(20)^, cognitive function before hospital admission, location before hospitalization, time from hospital admission to ICU entry, fiscal year of hospital admission, type of ICU admission, primary diagnosis, procedures on the day of ICU admission, and characteristics of the ICU. The *International Classification of Diseases, Tenth Revision*, codes used to define primary diagnosis are provided in Supplementary Table 2.

### Statistical analyses

Our primary approach for comparing ICU mortality between resource-rich ICUs vs. standard ICUs was to use a generalized estimating equation to determine the associations between ICU-level characteristics and the patient-level outcome, with ICUs as the clusters ^(21)^. We adjusted for all the ICU-level characteristics in Table 1 and patient-level characteristics in Table 2 as covariates. Differences and their 95% confidence intervals were calculated using generalized estimating equations with the identity link function and an equal-correlation structure model.

**Table 1.** ICU Characteristics.

	Resource-rich ICUs	Standard ICUs	
Characteristics	(n = 111)	(n = 347)	P-value
Annual ICU admissions, median (IQR)	641 (461-947)	499 (341-711)	<0.001
Number of hospital beds, median (IQR)	596 (490-798)	448 (340-594)	<0.001
Number of ICU beds, median (IQR)	10 (8-13)	8 (6-10)	<0.001
Teaching hospital, n (%)	97 (87)	307 (88)	0.76
Academic hospital, n (%)	46 (41)	37 (11)	<0.001
Number of dedicated nurses per ICU bed, median (IQR)	3.4 (2.9-4.0)	3.4 (2.9-4.0)	0.94
Dedicated pharmacist staffing, n (%)	39 (35)	64 (18)	<0.001
Dedicated physical therapist staffing, n (%)	9 (8)	15 (4)	0.12

Continuous variables are presented as medians and IQRs, whereas categorical variables are presented as numbers and percentages. Hospital characteristics were compared using Wilcoxon rank-sum test for continuous variables and chi-square test for categorical variables. ICU, intensive care unit; IQR, interquartile range

**Table 2.** Patient Characteristics.

	Resource-rich ICUs	Standard ICUs	
Characteristic	(n = 237,138)	(n = 552,492)	SMD
Age, years, median (IQR)	70 (60-78)	72 (62-79)	−13
Male, n (%)	143,493 (61)	337,273 (61)	−1.1
Body mass index at admission, kg/m^2^, n (%)
<18.5	29,465 (12)	69,152 (13)	−0.3
18.5-24.9	139,097 (59)	322,494 (58)	0.6
25.0-29.9	47,120 (20)	110,158 (20)	−0.2
≥30.0	11,405 (5)	26,204 (5)	0.3
Missing data	10,051 (4)	24,484 (4)	−0.9
Japan Coma Scale at admission, n (%)
Alert	201,020 (85)	448,656 (81)	9.5
Dizzy	19,007 (8)	53,680 (10)	−6
Somnolent	6,305 (3)	19,846 (4)	−5.4
Coma	10,806 (5)	30,310 (5)	−4.3
Charlson Comorbidity Index, median (IQR)	1.0 (0.0-2.0)	1.0 (0.0-2.0)	0.6
Cognitive function before admission, n (%)
No dementia	216,457 (91)	486,852 (88)	4.7
Mild dementia	13,889 (6)	42,547 (8)	−7.3
Moderate/severe dementia	6,792 (3)	23,093 (4)	−7.1
Location before hospitalization, n (%)
Home	217,750 (92)	506,331 (92)	0.7
Other hospital	16,058 (7)	32,298 (6)	3.8
Nursing home	3,330 (1)	13,863 (3)	−8
Time from admission to ICU entry, n (%)
On the day of admission	61,896 (26)	200,962 (36)	−22.3
After 1 day	37,225 (16)	92,542 (17)	−2.9
After 2-4 days	73,191 (31)	141,226 (26)	11.8
After >4 days	64,826 (27)	117,762 (21)	14.1
Fiscal year of admission, n (%)
2016	80,997 (34)	193,446 (35)	−1.8
2017	79,826 (34)	187,672 (34)	−0.6
2018	76,315 (32)	171,374 (31)	2.5
Type of ICU admission, n (%)
Elective surgery	131,450 (55)	245,299 (44)	22.2
Emergency surgery	32,059 (14)	83,522 (15)	−4.6
Non-operative	73,629 (31)	223,671 (40)	−19.8
Primary diagnosis, n (%)
Malignant neoplasms of digestive organs	28,925 (12)	74,423 (13)	−3.8
Other neoplasm	52,419 (22)	88,479 (16)	15.6
Congestive heart failure	29,284 (12)	63,949 (12)	2.4
Ischemic heart diseases	22,765 (10)	68,181 (12)	−8.8
Cerebrovascular diseases	15,161 (6)	51,601 (9)	−11
Aortic aneurysm and dissection	22,222 (9)	41,940 (8)	6.4
Other diseases of the circulatory system	10,651 (4)	29,671 (5)	−4.1
Diseases of the respiratory system	7,312 (3)	19,639 (4)	−2.6
Diseases of the digestive system	10,661 (4)	32,641 (6)	−6.4
Diseases of the connective tissue	9,218 (4)	17,016 (3)	4.4
Injury, poisoning, or external causes	8,290 (3)	23,084 (4)	−3.6
Miscellaneous	20,230 (9)	41,868 (8)	3.5
Procedures on the day of ICU admission, n (%)
Invasive mechanical ventilation	35,398 (15)	76,229 (14)	3.2
Dopamine	39,755 (17)	86,631 (16)	2.9
Dobutamine	32,756 (14)	47,670 (9)	16.5
Noradrenaline	68,044 (29)	121,150 (22)	15.6
Adrenaline	21,900 (9)	37,180 (7)	9.3
Vasopressin	3,976 (2)	5,911 (1)	5.2
Red blood cell transfusion	55,254 (23)	98,382 (18)	13.6
Fresh frozen plasma transfusion	39,278 (17)	64,069 (12)	14.3
Platelet transfusion	21,719 (9)	33,424 (6)	11.7
Renal replacement therapy	8,694 (4)	16,284 (3)	4
Extracorporeal membrane oxygenation	2,008 (1)	3,615 (1)	2.2

Continuous variables are presented as medians and IQRs, whereas categorical variables are presented as numbers and percentages. Patient characteristics were compared using SMDs. ICU, intensive care unit; SMD, standardized mean difference; IQR, interquartile range

### Subgroup analyses

We also compared ICU mortality between resource-rich ICUs and standard ICUs for seven patient subpopulations, according to the type of ICU admission (elective surgery, emergency surgery, and non-operative treatment), use of noradrenaline on the day of ICU admission, and use of invasive mechanical ventilation on the day of ICU admission.

### Sensitivity analyses

We performed an instrumental variable analysis to confirm the robustness of our findings and to address unmeasured confounders ^(22), (23)^. We then chose differential distance as the instrumental variable ^(22), (23)^. Differential distance was calculated as the difference between the driving distance from the patient’s residence to the nearest resource-rich ICU minus the driving distance from the patient’s residence to the nearest ICU of any type. We then created a binary instrumental variable by assigning patients to one of two groups: differential distance = 0 km (living near a resource-rich ICU) or differential distance > 0 km (living near a standard ICU). To confirm the validity of differential distance as an instrumental variable, we assessed whether differential distance was highly correlated with admission to a resource-rich ICU (F statistic > 10). We have also confirmed that the covariates were not associated with differential distance by calculating the standardized mean differences. We used a two-stage residual inclusion estimation framework for the instrumental variable analysis ^(24)^.

Continuous variables are presented as medians and interquartile ranges, whereas categorical variables are presented as numbers and percentages. Hospital characteristics were compared using the Wilcoxon rank-sum test for continuous variables and the chi-square test for categorical variables. Using Wilcoxon rank-sum tests and the chi-square tests for comparison of patient characteristics between the two ICU groups, all the p-values were almost zero because of the large sample size. Therefore, patient characteristics were compared using standardized mean differences, with absolute standardized differences >10% considered to denote imbalances between the two groups ^(25)^. All analyses were performed using Stata/MP 16.0 software (StataCorp, College Station, TX, USA). There were no missing data in this study. All reported p-values were two-sided, and p < 0.05 was considered to be statistically significant.

## Results

**Figure 1.**
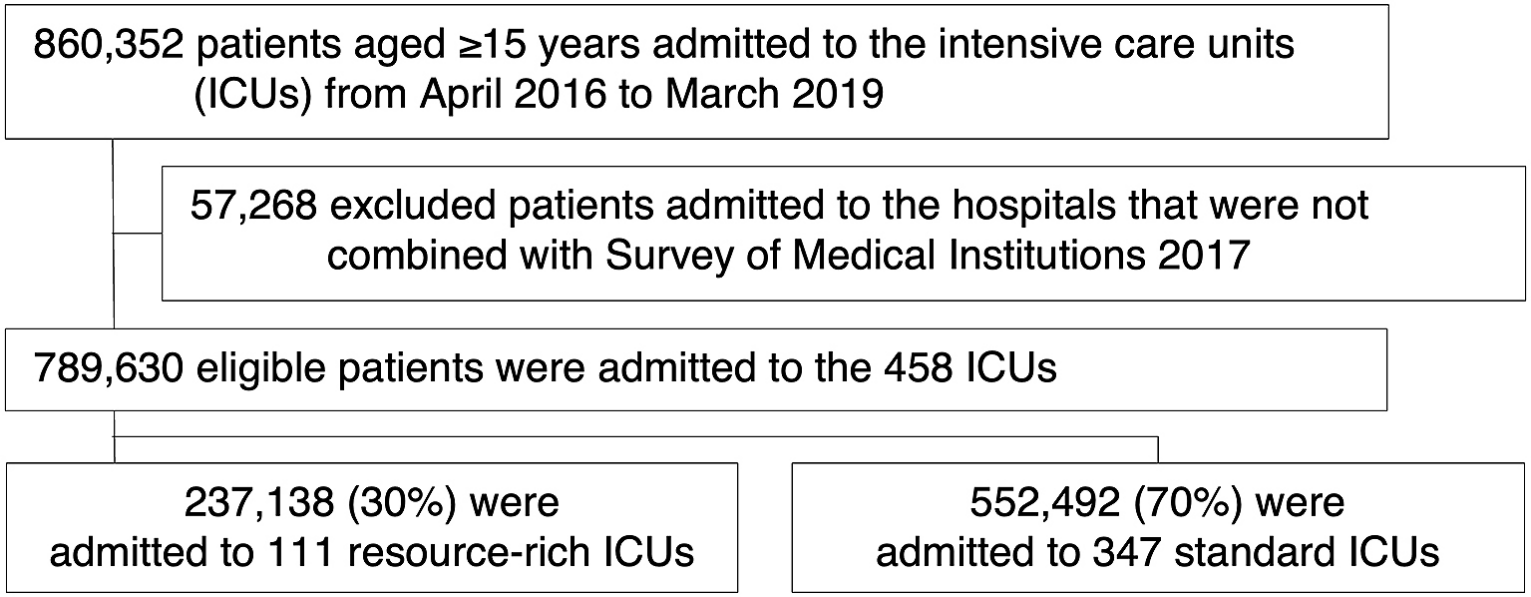
Flowchart of patient selection. ICU, intensive care unit

In total, 789,630 eligible patients from 458 ICUs were enrolled in this analysis. These 458 ICUs were equipped with a total 5,002 ICU beds, accounting for 70% (5,002/7,109) of all ICU beds in Japan in 2017. Of the eligible patients, 237,138/789,630 (30%) were admitted to resource-rich ICUs, whereas 552,492/789,630 (70%) were admitted to standard ICUs (Figure 1).

The characteristics of the 458 ICUs are shown in Table 1. The numbers of resource-rich ICUs and standard ICUs were 111/458 (24%) and 347/458 (76%), respectively. Resource-rich ICUs were more likely to have a large number of annual ICU admissions, to have large numbers of hospital and ICU beds, to be academic hospitals, and to have dedicated pharmacists working in the ICU, as compared with standard ICUs.

The characteristics of patients admitted to resource-rich ICUs and those admitted to standard ICUs are summarized in Table 2. Patients admitted to resource-rich ICUs were more likely to be younger, to be admitted to the ICU ≥2 days after hospital admission, to be admitted for elective surgery, to have other neoplasm as their primary diagnosis, and to receive dobutamine, noradrenaline, and transfusions. Patients admitted to standard ICUs were relatively likely to be admitted on the day of hospital admission and to be admitted for conditions other than surgery.

Crude ICU mortality was 3.6% (8443/237,138) among patients admitted to resource-rich ICUs and 4.3% (23,490/552,492) among those admitted to standard ICUs (Table 3). As per the generalized estimating equation analysis, patients cared for in resource-rich ICUs had significantly lower ICU mortality compared with those cared for in standard ICUs (difference, −0.4%; 95% confidence interval, −0.8%-0.0%).

**Table 3.** Difference in ICU Mortality between Resource-rich ICUs vs. Standard ICUs.

	ICU mortality		Generalized estimating equation		Instrumental variable	
Cohort	Resource-rich ICUs	Standard ICUs	Difference (95 % CI)	P-value	Difference (95 % CI)	P-value
**Overall analysis**	8443/237,138 (3.6)	23,490/552,492 (4.3)	−0.4 (−0.8 to 0.0)	0.047	−1.1 (−1.9 to −0.3)	0.005
**Subgroup analyses**
Type of ICU admission
Elective surgery	451/131,450 (0.3)	1006/245,299 (0.4)	0.0 (−0.1 to 0.0)	0.081	−0.1 (−0.2 to 0.1)	0.43
Emergency surgery	2602/32,059 (3.3)	2602/83,522 (3.1)	−0.2 (−0.7 to 0.2)	0.30	−0.4 (−1.3 to 0.5)	0.37
Non-operative	6934/73,629 (9.4)	19,882/223,671 (8.9)	−1.1 (−1.8 to −0.5)	0.001	−2.1 (−3.4 to −0.8)	0.002
Noradrenaline
Yes	4452/68,044 (6.5)	10,362/121,150 (8.6)	−1.0 (−1.7 to −0.3)	0.005	−1.6 (−2.9 to −0.4)	0.012
No	3,991/169,094 (2.4)	13,128/431,342 (3.0)	−0.2 (−0.7 to 0.2)	0.23	−1.0 (−1.8 to −0.2)	0.018
Invasive mechanical ventilation
Yes	4479/35,398 (12.7)	12,529/76,229 (16.4)	−1.3 (−2.5 to −0.1)	0.036	−2.3 (−4.4 to −0.1)	0.037
No	3,964/201,740 (2.0)	10,961/476,263 (2.3)	−0.1 (−0.3 to 0.2)	0.69	−0.5 (−1.1 to 0.0)	0.038

ICU, intensive care unit; CI, confidence interval

In the instrumental variable analysis, 188,537 patients were noted to be living near a resource-rich ICU, while 601,093 patients were living near a standard ICU. The binary instrumental variable of differential distance = 0 km vs. > 0 km was highly associated with admission to a resource-rich ICU (F statistic = 102,133). Excluding hospital characteristics, the other examined characteristics were well balanced between patients with a differential distance = 0 km and those with a differential distance of >0 km (Supplementary Table 3). The results of the instrumental variable analysis showed similar trends to those from the generalized estimating equations analyses in the overall cohort (Table 3).

In the subgroup analyses, the results in the subpopulations of patients with non-operative treatment, noradrenaline, and invasive mechanical ventilation in both the generalized estimating equation analysis and instrumental variable analysis showed a similar ICU mortality reduction with higher magnitude in the resource-rich ICU group compared with the results of the primary analysis (Table 3).

## Discussion

In this nationwide inpatient database study, care in resource-rich ICUs characterized by intensivist staffing, larger ICU area, and 24-hour medical engineer staffing was associated with lower ICU mortality compared with care in standard ICUs. This finding was observed to be more evident in subpopulations of patients with non-operative treatment, noradrenaline, and invasive mechanical ventilation. An instrumental variable analysis addressing unmeasured confounders ensured the robustness of our findings.

There are several possible reasons for this present study’s finding of lower ICU mortality among patients receiving care in resource-rich ICUs than among those receiving care in standard ICUs. The first is intensivist staffing: intensivists have the potential to improve patient care and outcomes in the ICU through their specialist knowledge of organ support therapies, extensive experience with critically ill cases, and higher compliance with evidence-based protocols ^(3)^. Previous observational studies have shown ICUs with intensivist staffing to be associated with lower mortality compared with ICUs without intensivist staffing ^(3), (4), (5)^. In Japan, there has been a nationwide shortage of intensivists, and the total number of intensivists certified in Japan by the Japanese Society of Intensive Care Medicine in 2012 was 935 ^(13)^. Conversely, the number of acute-care hospital beds relative to the population is about twice as high in Japan (about 700 per 100,000 population) as in other developed countries, decentralizing critical care resources in Japan ^(13), (14), (15), (16)^. Therefore, intensivist staffing in resource-rich Japanese ICUs may have contributed to the lower mortality observed among patients treated in these ICUs.

Second, the difference in hospital resources in resource-rich vs. standard ICUs may have contributed to the finding of lower mortality in resource-rich ICUs. Multidisciplinary team care has been reported to be associated with relatively low mortality ^(26)^. In our study, the percentage of ICUs with dedicated pharmacists and medical engineers was higher in resource-rich ICUs than in standard ICUs. In such resource-rich facilities, multidisciplinary care may have contributed to the relatively low mortality.

The above reasons may also explain the lower ICU mortality in the subpopulations of patients with non-operative treatment, noradrenaline, and invasive mechanical ventilation. These subsets of patients had higher morality and highest priority for ICU admission as recommended in the recent ICU admission guideline ^(27)^. Because surgical patients and non-critically ill patients had lower ICU mortality, the effect of resource-rich ICUs may have been weaker than that of standard ICUs. Additionally, because resource-rich ICUs have more limited availability and are more expensive than standard ICUs, appropriate use of these beds is preferred based on the findings of this present study.

To the best of our knowledge, this is the first nationwide study to demonstrate the potential benefit of resource-rich ICUs for patient outcomes in Japan. The main strength of our study was that the data included a large number of patients treated in the ICU and that the sample was nationally representative. Individual-patient observational designs with large numbers of covariates enable adjustment for measured individual risk. Our use of a generalized estimating equation approach considering ICU clustering enabled us to examine the impact of macro-level factors in ICUs on individual patients. Possible unmeasured confounders such as clinical severity, as measured with the well-known Acute Physiology and Chronic Health Evaluation clinical severity score, or the withholding or withdrawal of life-sustaining treatments, may still have caused confounding. However, we performed an instrumental variable analysis with an appropriate instrumental variable that seemed to satisfy the assumptions, suggesting the robustness of our findings.

As per the results of our study, resource-rich ICUs may be preferable for treating critically ill patients in Japan. Furthermore, to facilitate care in resource-rich ICUs, promoting and centralizing critical care resources would be desirable. We hope that our results will influence healthcare providers, hospital administrators, and policy makers to provide better critical care services in the future.

This present study had several limitations. First, this study showed the overall effect of treatment in hospitals with resource-rich ICUs and not the direct effect of the type of ICU. Therefore, future studies are required to assess the effect of organizational factors on mortality. Second, because of the nature of the analyzed administrative database, we were not able to obtain data on vital signs, laboratory findings, or risk adjustment scores such as the APACHE score, resulting in inadequate risk adjustment in our study. Finally, critical care systems vary around the world, and the results of this study may not be generalizable to other countries.

In conclusion, using a nationwide inpatient database, our study suggests that care in resource-rich ICUs with intensivist staffing, a larger ICU area, and 24-hour medical engineer staffing was associated with lower ICU mortality compared with care in standard ICUs. Intensivist staffing, larger ICU volume, and greater hospital resources in resource-rich ICUs may have contributed to the relatively low mortality in these ICUs. Further studies are required to assess the effect of organizational factors on mortality.

## Article Information

### Conflicts of Interest

None

### Sources of Funding

This work was supported by grants from the Ministry of Health, Labour and Welfare, Japan [grant numbers 21AA2007 and 20AA2005] and the Ministry of Education, Culture, Sports, Science and Technology, Japan [grant umber 20H03907].

### Author Contributions

HO, YS, and HY designed the research; HO, HM, and KF conducted the research; HO, YS, and HM analyzed the data; all authors wrote and revised the paper; and HO was primary responsible for the final content. All authors read and approved the final manuscript.

### Approval by Institutional Review Board (IRB)

The study was approved by the Institutional Review Board of the University of Tokyo (approval number 3501-3; December 25, 2017).

## Supplement

Supplementary Tables

## References

[1] Vincent JL, Rello J, Marshall J, et al. International study of the prevalence and outcomes of infection in intensive care units. JAMA. 2009;302(21):2323-9. 19952319 10.1001/jama.2009.1754

[2] Sakr Y, Moreira CL, Rhodes A, et al. The impact of hospital and ICU organizational factors on outcome in critically ill patients: results from the extended prevalence of infection in intensive care study. Crit Care Med. 2015;43(3):519-26. 25479111 10.1097/CCM.0000000000000754

[3] Kahn JM, Brake H, Steinberg KP. Intensivist physician staffing and the process of care in academic medical centres. Qual Saf Health Care. 2007;16(5):329-33. 17913772 10.1136/qshc.2007.022376 PMC2464974

[4] Parikh A, Huang SA, Murthy P, et al. Quality improvement and cost savings after implementation of the Leapfrog intensive care unit physician staffing standard at a community teaching hospital. Crit Care Med. 2012;40(10):2754-9. 22824939 10.1097/CCM.0b013e31825b26ef

[5] Pronovost PJ, Angus DC, Dorman T, et al. Physician staffing patterns and clinical outcomes in critically ill patients: a systematic review. JAMA. 2002;288(17):2151-62. 12413375 10.1001/jama.288.17.2151

[6] Carson SS, Stocking C, Podsadecki T, et al. Effects of organizational change in the medical intensive care unit of a teaching hospital: a comparison of 'open' and 'closed' formats. JAMA. 1996;276(4):322-8. 8656546

[7] Wilcox ME, Chong CA, Niven DJ, et al. Do intensivist staffing patterns influence hospital mortality following ICU admission? A systematic review and meta-analyses. Crit Care Med. 2013;41(10):2253-74. 23921275 10.1097/CCM.0b013e318292313a

[8] Sasabuchi Y, Yasunaga H, Matsui H, et al. The volume-outcome relationship in critically ill patients in relation to the ICU-to-hospital bed ratio. Crit Care Med. 2015;43(6):1239-45. 25756414 10.1097/CCM.0000000000000943

[9] Durairaj L, Torner JC, Chrischilles EA, et al. Hospital volume-outcome relationships among medical admissions to ICUs. Chest. 2005;128(3):1682-9. 16162775 10.1378/chest.128.3.1682

[10] Penoyer DA. Nurse staffing and patient outcomes in critical care: a concise review. Crit Care Med. 2010;38(7):1521-8. 20473146 10.1097/CCM.0b013e3181e47888

[11] The Japanese Society of Intensive Care Medicine, Committee of Social Insurance. Revision of the intensive care unit fee in 2014 [Internet]. 2014 [cited 2021 Mar 31]. Available from: https://www.jsicm.org/pdf/ICUsinnryou2014.pdf. Japanese.

[12] Ministry of Health, Labour and Welfare, Japan. Japan Ministry of Health, Labour and Welfare Statistical Surveys 2017 [Internet]. 2017 [cited 2021 Mar 31]. Available from: https://www.mhlw.go.jp/stf/seisakunitsuite/bunya/open_data.html. Japanese.

[13] Shime N. Clinical and investigative critical care medicine in Japan. Intensive Care Med. 2016;42(3):453-5. 26762107 10.1007/s00134-015-4165-7

[14] The Japanese Society of Intensive Care Medicine, Committee of Japanese ICU Evaluation. Influence of staffing and administrative policy of ICU on patient outcome. J Jpn Soc Intensive Care Med. 2011;18(2):283-94.

[15] Uchino S. Are Japanese ICUs properly utilized? J Jpn Soc Intensive Care Med. 2011;17(2):141-4.

[16] Japanese Society of Intensive Care Medicine, Committee of Japanese ICU Evaluation, Imanaka Y, Hayashida K, et al. Physician staffing and patient outcome in Japanese ICUs. J Jpn Soc Intensive Care Med. 2010;17(2):227-32.

[17] Yasunaga H. Real world data in Japan: chapter II the diagnosis procedure combination database. Ann Clin Epidemiol. 2019;1(3):76-9.

[18] Yamana H, Moriwaki M, Horiguchi H, et al. Validity of diagnoses, procedures, and laboratory data in Japanese administrative data. J Epidemiol. 2017;27(10):476-82. 28142051 10.1016/j.je.2016.09.009 PMC5602797

[19] Shigematsu K, Nakano H, Watanabe Y. The eye response test alone is sufficient to predict stroke outcome―reintroduction of Japan Coma Scale: a cohort study. BMJ Open. 2013;3(4):e002736. 10.1136/bmjopen-2013-002736 PMC3641437 23633419

[20] Quan H, Li B, Couris CM, et al. Updating and validating the Charlson comorbidity index and score for risk adjustment in hospital discharge abstracts using data from 6 countries. Am J Epidemiol. 2011;173(6):676-82. 21330339 10.1093/aje/kwq433

[21] Hubbard AE, Ahern J, Fleischer NL, et al. To GEE or not to GEE: comparing population average and mixed models for estimating the associations between neighborhood risk factors and health. Epidemiology. 2010;21(4):467-74. 20220526 10.1097/EDE.0b013e3181caeb90

[22] Baiocchi M, Cheng J, Small DS. Instrumental variable methods for causal inference. Stat Med. 2014;33(13):2297-340. 24599889 10.1002/sim.6128 PMC4201653

[23] Garabedian LF, Chu P, Toh S, et al. Potential bias of instrumental variable analyses for observational comparative effectiveness research. Ann Intern Med. 2014;161(2):131-8. 25023252 10.7326/M13-1887

[24] Terza JV, Basu A, Rathouz PJ. Two-stage residual inclusion estimation: addressing endogeneity in health econometric modeling. J Health Econ. 2008;27(3):531-43. 18192044 10.1016/j.jhealeco.2007.09.009 PMC2494557

[25] Austin PC. Using the standardized difference to compare the prevalence of a binary variable between two groups in observational research. Commun Stat Simul Comput. 2009;38(6):1228-34.

[26] Kim MM, Barnato AE, Angus DC, et al. The effect of multidisciplinary care teams on intensive care unit mortality. Arch Intern Med. 2010;170(4):369-76. 20177041 10.1001/archinternmed.2009.521 PMC4151479

[27] Nates JL, Nunnally M, Kleinpell R, et al. ICU admission, discharge, and triage guidelines: a framework to enhance clinical operations, development of institutional policies, and further research. Crit Care Med. 2016;44(8):1553-602. 27428118 10.1097/CCM.0000000000001856

