# Supplementary material for: Resource-rich Intensive Care Units vs. Standard Intensive Care Units on Patient Mortality: A Nationwide Inpatient Database Study
Source: JMA J. 2021 Sep 27;4(4):397–404. doi: 10.31662/jmaj.2021-0098 (PMC8580699; doi:10.31662/jmaj.2021-0098)
Supplement: Supplementary file 1 — Supplementary Tables [file 2433-3298-4-4-0397-s001.pdf]

List of Supplementary Data

Supplemental Tables:

Supplemental Table 1. Japanese medical procedure codes used to define resource-rich ICUs and standard ICUs

Supplemental Table 2. *ICD-10* codes used to define primary diagnosis

Supplemental Table 3. Characteristics and outcomes of patients by differential distance (= 0 km vs. > 0 km) for the instrumental variable analysis

Supplemental Table 1. Japanese medical procedure codes used to define the resource-rich ICUs and standard ICUs

Name	Code	Description	Cost per day in USD*
Resource-rich ICU	A3011	ICU management fee 1	1,292
Resource-rich ICU	A3012	ICU management fee 2	1,292
Standard ICU	A3013	ICU management fee 3	882
Standard ICU	A3014	ICU management fee 4	882

*Cost per day is shown for the first 7 days after ICU admission.

*We assumed 1 US dollar was equivalent to 110 Japanese yen.

ICU, intensive care unit; USD, United States Dollars

Supplemental Table 2. *ICD-10* codes used to define primary diagnosis

Name	*ICD-10* codes
Malignant neoplasms of digestive organs	C15–C26
Other neoplasm	C00–C14, C30–D48
Congestive heart failure	I01, I05–I09, I110, I130, I132, I30–I43, I50–I52
Ischemic heart diseases	I20–I25
Cerebrovascular diseases	I60–I69
Aortic aneurysm and dissection	I71
Other diseases of the circulatory system	I02–I04, I10, I119, I12, I131, I139, I15, I26–I28, I44–I49, I70, I72–I99
Diseases of the respiratory system	J00–J99
Diseases of the digestive system	K00–K93
Diseases of the connective tissue	M00–M99
Injury, poisoning, or external causes	S00–T98
Miscellaneous	*ICD-10* codes other than those listed above

ICD-10, International Classification of Diseases, Tenth Revision

Supplemental Table 3. Characteristics and outcomes of patients by differential distance (= 0 km vs. > 0 km) for the instrumental variable analysis

	Differential distance = 0 km (n = 188,537)	Differential distance > 0 km (n = 601,093)	SMD
Admission to a resource-rich ICU, n (%)	120,821 (64)	116,317 (19)	101.8
Age, years, median (IQR)	71 (61–79)	71 (62–79)	−1.6
Male, n (%)	114,604 (61)	366,162 (61)	−0.3
Body mass index at admission, kg/m^2, n (%)			
< 18.5	23,424 (12)	75,193 (13)	−0.3
18.5–24.9	108,935 (58)	352,656 (59)	−1.8
25.0–29.9	37,328 (20)	119,950 (20)	−0.4
≥ 30.0	9,072 (5)	28,537 (5)	0.3
Missing	9,778 (5)	24,757 (4)	5.1
Japan Coma Scale at admission, n (%)			
Alert	154,968 (82)	494,708 (82)	−0.3
Dizzy	17,374 (9)	55,313 (9)	0
Somnolent	6,183 (3)	19,968 (3)	−0.2
Coma	10,012 (5)	31,104 (5)	0.6
Charlson comorbidity index, median (IQR)	1.0 (0.0–2.0)	1.0 (0.0–2.0)	1.6
Cognitive function before admission, n (%)			
No dementia	168,933 (90)	534,376 (89)	2.7
Mild dementia	12,808 (7)	43,628 (7)	−1.8
Moderate/severe dementia	6,796 (4)	23,089 (4)	−1.2
Location before hospitalization, n (%)			
Home	172,728 (92)	551,353 (92)	−0.4
Other hospital	11,891 (6)	36,465 (6)	1
Nursing home	3,918 (2)	13,275 (2)	−0.9
Time from admission to ICU entry, n (%)			
On the day of admission	63,068 (33)	199,790 (33)	0.5
After 1 day	30,365 (16)	99,402 (17)	−1.2
After 2–4 days	51,650 (27)	162,767 (27)	0.7
After > 4 days	43,454 (23)	139,134 (23)	−0.2
Fiscal year of admission, n (%)			
2016	65,631 (35)	208,812 (35)	0.2
2017	63,389 (34)	204,109 (34)	−0.7
2018	59,517 (32)	188,172 (31)	0.6
Type of ICU admission, n (%)			
Elective surgery	90,536 (48)	286,213 (48)	0.8
Emergency surgery	26,523 (14)	89,058 (15)	−2.1
Non-operative	71,478 (38)	225,822 (38)	0.7
Primary diagnosis, n (%)			
Malignant neoplasms of digestive organs	23,108 (12)	80,240 (13)	−3.3
Other neoplasm	34,185 (18)	106,713 (18)	1
Congestive heart failure	23,687 (13)	69,546 (12)	3.1
Ischemic heart diseases	22,082 (12)	68,864 (11)	0.8
Cerebrovascular diseases	14,594 (8)	52,168 (9)	−3.4
Aortic aneurysm and dissection	16,015 (8)	48,147 (8)	1.8
Other diseases of the circulatory system	9,288 (5)	31,034 (5)	−1.1

Diseases of the respiratory system	6,421 (3)	20,530 (3)	−0.1
Diseases of the digestive system	9,801 (5)	33,501 (6)	−1.7
Diseases of the connective tissue	6,764 (4)	19,470 (3)	1.9
Injury, poisoning, or external causes	7,341 (4)	24,033 (4)	−0.5
Miscellaneous	15,251 (8)	46,847 (8)	1.1
Procedures on the day of ICU admission, n (%)			
Invasive mechanical ventilation	29,306 (16)	82,321 (14)	5.2
Dopamine	30,532 (16)	95,854 (16)	0.7
Dobutamine	21,101 (11)	59,325 (10)	4.3
Noradrenaline	50,173 (27)	139,021 (23)	8.1
Adrenaline	16,508 (9)	42,572 (7)	6.2
Vasopressin	2,859 (2)	7,028 (1)	3
Red blood cell transfusion	38,049 (20)	115,587 (19)	2.4
Fresh frozen plasma transfusion	25,897 (14)	77,450 (13)	2.5
Platelet transfusion	13,911 (7)	41,232 (7)	2
Renal replacement therapy	6,880 (4)	18,098 (3)	3.6
Extracorporeal membrane oxygenation	1,561 (1)	4,062 (1)	1.8
Hospital characteristics			
Annual ICU admissions, median (IQR)	764 (565–1140)	718 (499–1158)	10.5
Number of hospital beds, median (IQR)	596 (480–812)	560 (400–733)	25.6
Number of ICU beds, median (IQR)	10 (8–16)	10 (7–14)	10.4
Teaching hospital, n (%)	154,258 (82)	495,106 (82)	−1.4
Academic hospital, n (%)	64,763 (34)	164,150 (27)	15.3
Number of dedicated nurses per ICU bed, median (IQR)	3 (3–4)	3 (3–4)	−0.3
Dedicated pharmacist staffing, n (%)	67,719 (36)	156,941 (26)	21.3
Dedicated physical therapist staffing, n (%)	15,416 (8)	37,570 (6)	7.5
Outcome			
ICU mortality, n (%)	7,674 (4.1)	24,259 (4.0)	0.2

ICU, intensive care unit; SMD, standardized mean difference; IQR, interquartile range
